# Theoretical Evaluation of the Influence of Molecular Packing Mode on the Intramolecular Reorganization Energy of Oligothiophene Molecules

**DOI:** 10.3390/polym10010030

**Published:** 2017-12-27

**Authors:** Bing Zhang, Yingxue Xu, Lilin Zhu, Shijie Zhou, Yinjie Liao, Kaixuan Zhou, Jianxi Yao, Songyuan Dai

**Affiliations:** 1 Beijing Key Laboratory of Energy Security and Clean Utilization, North China Electric Power University, Beijing 102206, China; jianxiyao@ncepu.edu.cn; 2 Beijing Key Laboratory of Novel Thin Film Solar Cells, North China Electric Power University, Beijing 102206, China; 3 School of Renewable Energy, North China Electric Power University, Beijing 102206, China; melody.xu213@gmail.com (Y.X.); zllamber@163.com (L.Z.); sjwzzsj@126.com (S.Z.); lyj_1633@163.com (Y.L.); kaixuan.z1029@gmail.com (K.Z.)

**Keywords:** oligothiophene molecules, molecular dynamics, quantum mechanics, molecular mechanics/quantum mechanics, packing mode, reorganization energy

## Abstract

Accurate determination of the relationships among packing mode, molecular structure and charge transfer mobility for oligothiophene analogues has been significantly impeded, due to the lack of crystal structure information. In the current study, molecular dynamics (MD) were used to investigate the packing mode of non-, methyl- and ethyl-substituted poly(3-alkylthiophenes) (P3ATs). Obvious conformational changes were observed when comparing the packed and isolated oligothiophene molecules, indicating the important influence of packing mode on the geometric structures of these materials. Considering the crucial role played by reorganization energy (RE) in the charge transfer process, both quantum mechanics (QM) and quantum mechanics/molecular mechanics (QM/MM) were performed to examine the impact of different conformations on energy. Our simulations revealed that the geometric structures have distinct effects on the RE. Our data suggest that MD could give a reliable packing mode of oligothiophene analogues, and that QM/MM is indispensable for precisely estimating RE.

## 1. Introduction

Thiophene-based materials have attracted much attention in organic photovoltaic cells, due to the large π-conjugation and excellent chemical and physical properties [1]; regioregular poly(3-hexylthiophene) (P3HT) is even used as a model polymer for research in organic solar cells. [2,3]. Owing to the excellent charge transfer properties and structural simplicity, poly(3-alkylthiophene) (P3AT) based oligothiophene analogues have been considered ideal targets to investigate mechanisms of charge transfer processes [4,5,6,7,8]. Although these mechanisms are still not fully understood, Marcus theory has been used extensively to study the factors that influence the charge motilities [9], and two key parameters, i.e., the transfer integral (intermolecular electronic coupling), and the reorganization energy λ (RE, the local electron–phonon coupling) have been shown to determine self-exchange charge transfer rates. The reorganization energy of self-exchange charge transfer reaction in a hole-hopping material is defined as the sum of the geometrical relaxation energies of a molecule upon going from the neutral state to the charge state geometry, and the neighboring molecules upon going from the inverse process [10]. RE can, therefore, be divided into two terms: internal RE and solvent RE. Solvent RE is related to the polarization of the surrounding medium, and has been discovered to be small. Generally, the larger transfer integral and lower RE can lead to higher charge carrier mobility. The rational design of molecules with small λ has therefore been deemed as a feasible method to improve carrier mobility. However, it has been shown that the RE of organic semiconductors can be affected by the molecule conformation. 

Molecular packing is a crucial factor for charge transport properties. The interaction between neighboring molecules can vary greatly for different packing modes, which results in different transport efficiencies and anisotropies [11]. Due to limitations of current experimental techniques in determining microscopic structure and charge mobility in semiconducting polymers, it is difficult to elucidate the influence of sample morphology on charge transport. Fortunately, accurate theoretical simulations can help to explain, qualitatively or quantitatively, the relationship between polymer structures and their charge transport properties, thereby also aiding in explicating results from experiments [12,13]. In the recent years, the model polymer P3HT was investigated from many aspects. Bhatta et al. [14] developed an ab initio-based force field, and validated by MD, simulations of packing of crystalline P3HTs. The density, the melting temperature and surface tension derived from the theoretical simulations were all consistent with experimental values. Detailed MD simulations were carried out to simulate the pure amorphous phases of regioregular P3HT, but only a transition from a pure amorphous liquid-like phase to a semicrystalline state was found [15]. However, the influence of molecular packing mode on the charge mobility of the oligothiophene molecules, as far as we know, have not reported yet. 

Recently, Li et al. [16] reported the obvious influences of molecular packing on the RE of four commonly used small-molecule organic semiconductors, suggesting that to avoid large deviations in mobility estimations, molecular packing should be taken into account when calculating the intramolecular RE. These data indicate that the significance of this influence depends on the degree of reduced conformational change, compared to that of isolated molecules. 

X-ray diffraction experiments showed that the packing mode of different lengths of P3AT presented a herringbone pattern [17,18,19], where the hexyl substituted poly(3-hexylthiophene) (P3HT) tends to form stacking plates [20,21,22]. In all of the crystal structures, the poly-thiophene ring motif is planar, however, our preliminary results and the quantum mechanics simulations from Yang et al. [23] show bow-shaped thiophene compounds. 

On one hand, the backbone of P3ATs is made only of isolated rings and linear side chains, which gives the polymer chains significant freedom to sample conformational space; on the other hand, the influence of packing mode on the single molecule conformations of oligothiophenes remains unclear, due to a lack of crystal structure information for different lengths and various substituted oligothiophenes. Consequently, studies into the relationships between spatial packing, molecular structure, and charge transfer mobility have been significantly restricted. 

Our previous work has demonstrated that long time molecular dynamics simulations (MD) can ideally reproduce the packing mode of different lengths of P3ATs [24]. In the current work, the packing modes of methyl- and ethyl-substituted 3T–8T P3ATs were firstly explored via MD simulations, and then conformations of non-, methyl- and ethyl-substituted 3T–8T P3ATs from the MD simulations and quantum mechanics (QM) optimizations were compared and analyzed. The RE of the 18 molecules with different geometric structures was computed by both quantum mechanics and quantum mechanics/molecular mechanics (QM/MM) to reveal the influence of molecule packing on the intramolecular RE.

## 2. Materials and Methods

### 2.1. Molecular Dynamics of the Packing Modes

In the current study, the 18 initial structures of the methyl- and ethyl-substituted 3T–8T P3AT molecules were constructed using the graphic interface of the Material Studio software suite (Accelrys Software Inc., San Diego, CA, USA) [25]. Optimization of the structures was achieved with the Gaussian09 package (Gaussian, Inc., Wallingford, CT, USA) [26] at the HF/6-31G* theoretical level. Electrostatic potentials (ESP) were then generated with Merz–Singh–Kollman van der Waals parameters [27]. Fitting of the charges to the ESP was conducted with the restrained ESP (RESP) program (University of California, San Francisco, CA, USA) [28] of the AMBER package [29]. GAFF [29] force field parameters and RESP partial charges were assigned by using the ANTECHAMBER module (University of California, San Francisco, CA, USA). The starting aggregation models were established with a systematic density of 2 g/cm^3^ on the amorphous cell module of the Material Studio suite (Accelrys Software Inc., San Diego, CA, USA), based on the force field parameters in a consistent valence force field (CVFF) [30]. 

**Figure 1:**
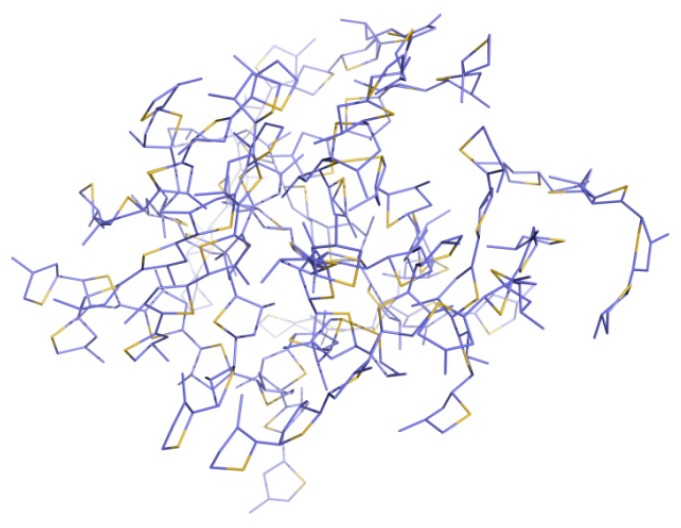
The aggregation of 20 methyl-substituted 5T P3ATs.

To make the simulation more consistent with real cases, each system consisted of amorphous structures containing 20 oligothiophene molecules (Figure 1). The aggregation of 20 methyl-substituted 5T P3ATs was taken as an example. The MD simulations were carried out with the sander module provided in the dynamic software package Amber14 (University of California, San Francisco, CA, USA) [29]. With periodic boundary conditions, the 18 systems were range-limited in periodic water boxes of 36.00 Å × 34.26 Å × 31.35 Å, 34.99 Å × 37.80 Å × 39.27 Å, 43.21 Å × 36.76 Å × 37.19 Å ×, 41.55 Å × 41.43 Å × 43.56 Å, 53.78 Å × 46.30 Å × 46.88 Å, 49.93 Å × 53.03 Å × 50.63 Å, 34.99 Å × 35.78 Å × 31.35 Å, 35.89 Å × 36.45 Å × 38.62 Å, 42.11 Å × 38.31 Å × 41.93 Å, 45.56 Å × 47.28 Å × 43.56 Å, 51.73 Å × 51.64 Å × 53.45 Å, 58.32 Å × 54.19 Å × 55.36 Å, respectively. An Ewald particle mesh [31] was adopted to calculate long-range electrostatic interactions. Energy optimization of each system was made within 1500 steps in total. In the former 500 steps, the steepest descent method was used and after the 500 steps, a conjugate gradient method was utilized. The systems were then heated to 298 K within 100 ps, so as to carry out the MD simulation under constant temperature and pressure in 1 fs for 100 ns. In this process, temperature and pressure were controlled by a weak-coupling algorithm and an isotropic position scaling method [32], respectively. The data processing and analysis was made using cpptraj [33] module in Amber14 (University of California, San Francisco, CA, USA).

### 2.2. QM and QM/MM Calculations of Reorganization Energy

RE λ was computed as half the energy difference between the vertical excitation and florescence energies as described before [16,34]. For the pure quantum mechanics calculations of λ, two separate optimizations of the structure of the isolated molecular cation in the ground state (*S*_0_) and in the first singlet excited state (*S*_1_) were firstly performed. Single-point calculations of the *S*_0_ → *S*_1_ excitation energy of the cation at the ground state geometry Δ*E*(*S*_0_), and at the *S*_1_ excited state geometry Δ*E*(*S*_1_) were then performed, i.e.,

λ = [Δ*E*(*S*_0_) − Δ*E*(*S*_1_)]/2. (1)

All the geometry optimization computations were carried out using the Gaussion09 program at the B3lyp/6-31G and B3lyp/6-311G** levels, respectively. 

**Figure 2:**
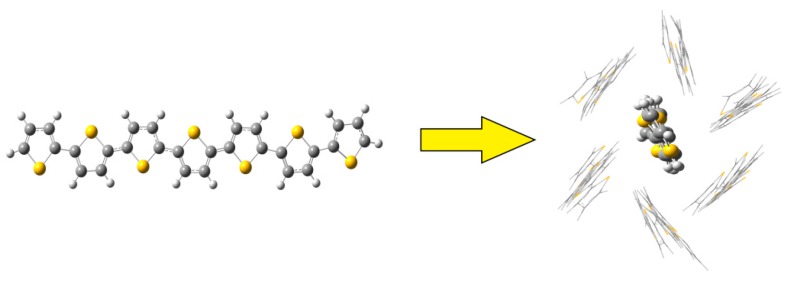
The schematic illustration of the QM/MM methodology.

The packing effect on the RE was estimated using the QM/MM method by the ONIOM module [35] in the Gaussian 09 program (Gaussian, Inc., Wallingford, CT, USA). In the QM/MM approach, the QM was only applied to the center molecule, while the MM computations of the surrounding molecules were conducted with the general Amber force field (GAFF) [29], as depicted in Figure 2. During the calculations, all the ambient oligothiophene molecules were fixed, and the center molecule was free to move. The same methodology and theory levels for the QM section were used as described in the pure QM calculations for precise comparisons. 

## 3. Results

### 3.1. Packing Mode of Different Length and Different Substituted P3AT

**Figure 3:**
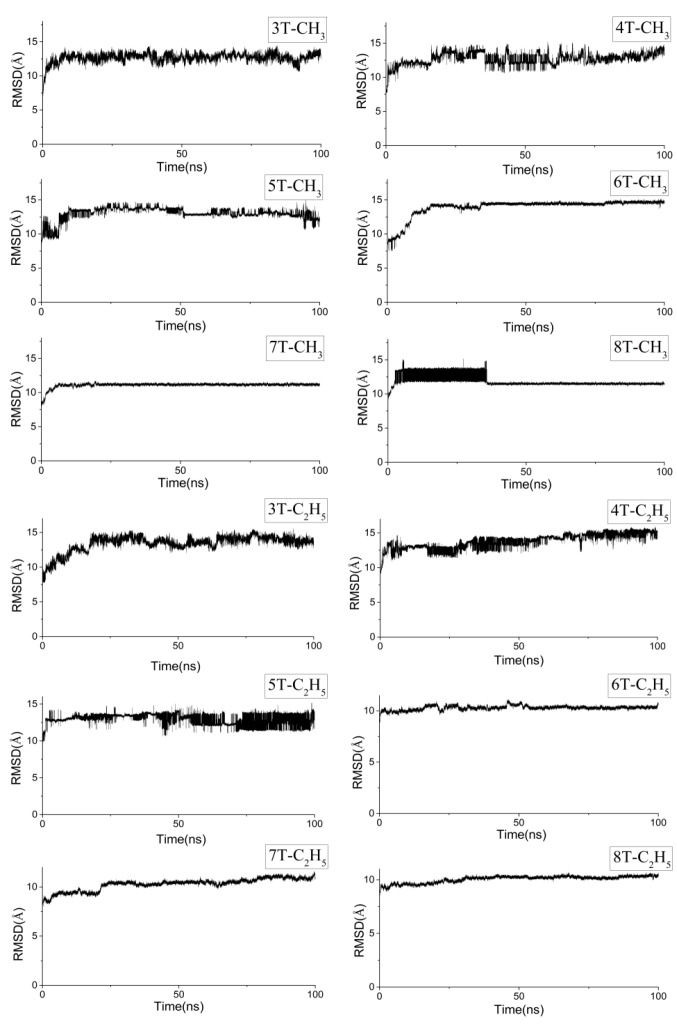
The root mean square deviations (RMSDs) of all the systems during the dynamic processes.

The packing mode of non-substituted P3AT by our MD simulations has been reported elsewhere [22], only the results of methyl- and ethyl-substituted P3ATs are discussed here. Figure 3 shows the variations of the root mean square deviations (RMSDs) of all the systems during the dynamic processes. As seen, the curves for each of the 12 systems stabilized after a certain time-span of fluctuations, indicating the molecular packing processes from the starting amorphous mode to a certain arrangement pattern. 

**Figure 4:**
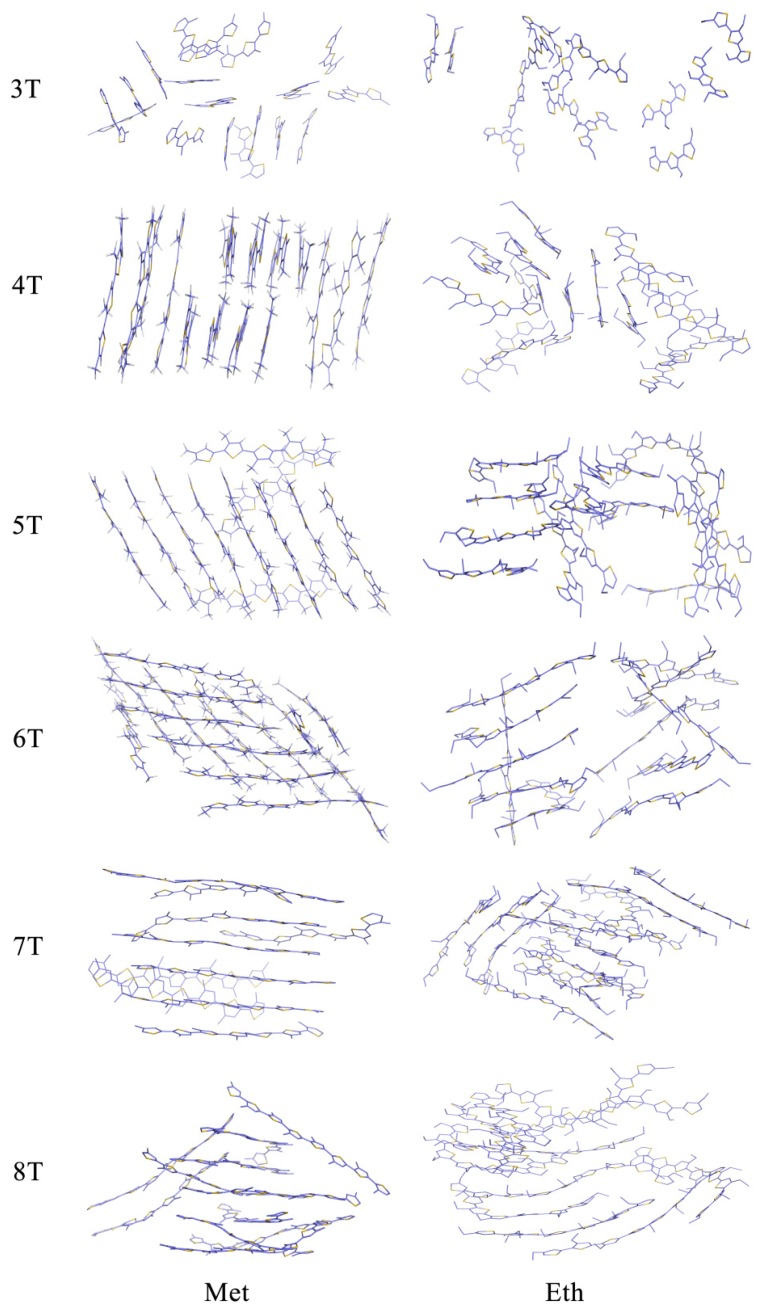
The packing pattern of the methyl- and ethyl-substituted P3AT molecules, respectively.

In contrast to data generated from our previous theoretical models [24] and the crystal structures [17,18,19,36] of the herringbone packing pattern of the non-substituted P3AT chains, the methyl- and ethyl-substituted oligothiophene molecules were arranged in parallel during the dynamic equilibrium, which ultimately led to a stacking plate packing mode, as depicted in Figure 4. Compared with the non-substituted structure [19], both the methyl- and ethyl-substituted 3T P3AT were less closely-packed, which was caused by side-chain replacement. However, with an increase in the main poly-thiophene chain length, all of the oligothiophene molecules appear to have a close-packed arrangement, although side-chain substitution is unfavorable for close-packing, the negative influence from which could be overcome by the increase in the main chain length. From the figure, it is also clear that the methyl- and ethyl-substituted poly-thiophene ring main chain mainly shows a flattened conformation, which is consistent with our simulation results of the non-substituted P3AT molecules [24].

**Figure 5:**
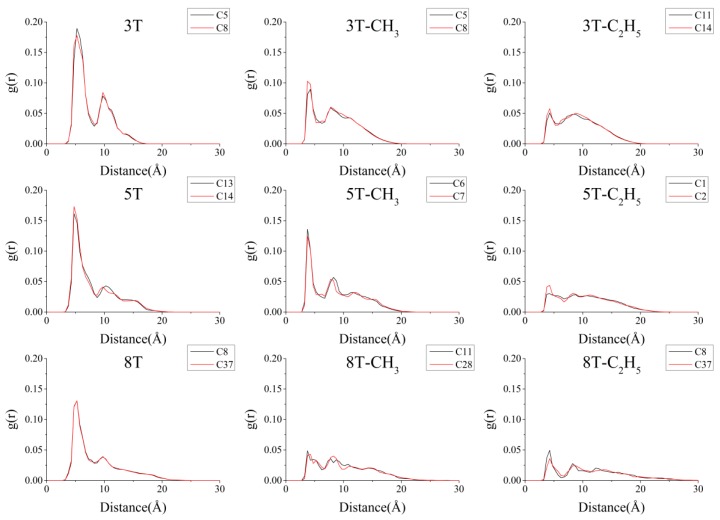
Radial distribution function between the carbon atoms in the center of the main chains of non-, methyl-, and ethyl-substituted 3T, 5T, and 8T molecules, respectively.

The radial distribution functions (RDFs) of selected carbon atoms in the center of the main-chain of the non-, methyl-, and ethyl-substituted 3T, 5T, and 8T P3ATs were computed to quantitatively describe the atomic packing and thiophene ring arrangement. In Figure 5, the non-substituted P3ATs interchain C–C RDFs exhibit strong peaks that indicate well-ordered nanostructure of these chains. It is clear that the peak intensity gets weaker and weaker in the methyl- and ethyl-substituted chains in all the 3T, 5T, and 8T cases, which means less compact packing modes, and is consistent with the qualitative results in Figure 4.

### 3.2. Different Conformations from QM, MD, and QM/MM Simulations

**Figure 6:**
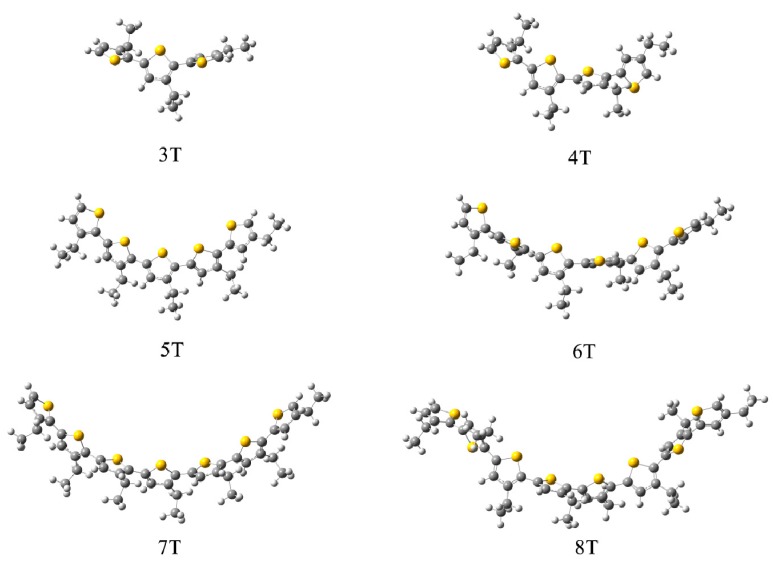
The QM optimized curved conformation of the ethyl-substituted P3ATs.

To precisely estimate the RE, the structures from either X-ray experiments or MD simulations had to first be optimized. Single isolated molecules were optimized by QM without any restrictions, and well packed molecules were managed via QM/MM, as described in the Section 2.2. In the case of QM optimizations, all the poly-thiophene main chains appear to have various degrees of curvature. The conformation of ethyl-substituted P3ATs is shown as an example in Figure 6. The torsion angles between the neighboring thiophene rings and the degrees of curvature of all the systems are given in Table 1 to illustrate the conformational deviation from planarity. As discussed, in both the crystallography data and the MD simulation results, the conformation of the oligothiophene molecules in the well packed groups was found to be planar, as shown in Figure 4 and listed in Table 1. From both the figure and the table, it is obvious that the curvature of the isolated candidates from the QM method is much larger than that of the packed structure from the QM/MM calculations. Taking the non-, methyl- and ethyl-substituted 8T analogues as an example, the curvature from the QM method was 0.02687, 0.07546, and 0.10528, respectively, whilst that from the QM/MM method was 0.00008, 0.00854, and 0.019833, respectively. The smaller curvature relates to less curved conformation.

From the torsion angle values in Table 1, it can be seen that with increasing side chain length, the distortion between the neighboring rings also increases. For example, in the case of 8-thiophene-ring chains, QM and QM/MM torsion angles for the non-, methyl-, ethyl-substituted molecules were 149.82°, 122.32°, 114.23°, and 175.74°, 176.07°, 174.78°, respectively. The QM/MM results reveal much less effect of the side chain on the planarity of the poly-thiophene derivatives. The straight and planar conformations could be further confirmed by the consistency with the previous report about the non-substituted P3AT and P3HT main chain conformation [17,18,19,37,38]. The phenomenon also proves the influence of the packing on the conformation of the oligothiophene structures.

### 3.3. Molecular Packing Influences on the Reorganization Energy

The RE of the 6 non-substituted oligothiophene molecules was firstly calculated by both the QM and QM/MM methods to explore the effects originating from the curved and flat conformations. The energy values are listed in Table 2. It can be seen from the first two columns of Table 2 that the molecular packing has a great influence on the calculated intramolecular RE. When the molecular packing was considered, the RE was about 15–20% lower than that of the isolated candidates. The RE of the methyl- and ethyl-substituted candidates was then calculated when the packing mode was included, to investigate the influence of conformational variations on the λ values. As can be observed in the table, with increasing side chain length, the λ values were also shown to increase. The increment between the methyl- and non-substitutions, and the ethyl- and non-substitutions is 1.0–4.5 kcal/mol and 1.4–5.3 kcal/mol, respectively. This is also consistent with the data in Figure 5, in which less close packing modes of the bigger side chain replacements always leads to lower RDFs. At the same time, it is evident that with the elongation of the poly-thiophene main chain, the RE decreases. However, it is observed that the presence of substitutions increases the RE by a greater amount for short systems (3T) compared to longer systems (8T). By taking a closer look at the data in Figure 5, the averaged RDF value of the non-substituted 3T, 5T, and 8T chains is 0.041, 0.030, and 0.020, respectively; that of the methyl- and ethyl-substituted 3T, 5T, and 8T molecules is 0.027, 0.024, 0.013 and 0.023, 0.014, 0.009, respectively. The reduction of the averaged RDFs from non- to ethyl-substituted 3T, 5T, and 8T molecules is 0.018, 0.014, and 0.011, respectively. 

## 4. Discussion

Although pure poly-thiophene rings normally adopt a herringbone packing pattern, the simulation results clearly demonstrate that all the substituted P3ATs aggregate in a stacking plate form, indicating the important influence of the substitution on the packing mode. Furthermore, the conformation of the chains results in very different geometric structures when the packing mode is taken into consideration. Compared to the bowed conformations from the QM calculations, the QM/MM optimizations show a straight and planar conformation. From Table 1, it is obvious that, for the QM optimized isolated molecules, the bigger the substitution group, the more twisted the torsion angle between the neighboring thiophene rings, i.e., the bigger the substitution, the bigger influence on the planarity of the oligothiophene molecules. Considering the RE calculations as shown in the first two columns in Table 2, our data strongly suggests that when calculating systems with bigger substitutions, more emphasis should be given to choosing the most appropriate theoretical methodology, to obtain conformations that are as close as possible to the experimental packing mode. According to the Marcus theory, smaller RE leads to larger hopping rates, which means the charge mobility could be underestimated if the molecular packing is overlooked.

When the environment of the isolated molecules was considered, all the optimized structures are near planar, and the data in Table 1 clearly demonstrate that, irrespective of the planarity or the torsion angles, with the elongation of the poly-thiophene main chain, the RE decreases. These changes reflect the fact that a larger π-conjugation system is always more stable, and benefits the charge transport.

## 5. Conclusions

In summary, the packing modes of the non-, methyl-, and ethyl-substituted P3ATs were firstly simulated by MDs, and the RE of the P3ATs was calculated in the cases of single isolated and packed molecules. Our simulations show significant influence of the packing on the conformation of all the structures. When the packing pattern was considered, all the molecules show a flat conformation. Compared with the curved and twisted geometries, the flat conformation gives a smaller RE, and would eventually affect the charge mobility estimation. A longer main chain with more monomer units expands the π-electron delocalization, and decreases RE as well. Our simulations also reveal that the bigger substitution results in larger RE. Further investigations, therefore, need be done to find the optimal combination of a suitable size of substitution on a suitable length of main chain for an optimal RE value.

Our work proves that MD simulation is a reliable tool to locate proper packing modes of the oligothiophene molecules, and that QM/MM calculations are necessary and precise for the consideration of molecular packing, especially when big substitution groups are attached on the main chains. 

## Figures and Tables

**Table 1:** The torsion angles between the neighboring thiophene rings and the degrees of curvature.

System	Method	Curvature	Torsion Angles (°)
3T	QM	0.03294	148.67
	QM/MM	0.02813	167.12
4T	QM	0.02964	149.18
	QM/MM	0.01035	177.91
5T	QM	0.03098	149.44
	QM/MM	0.00430	172.87
6T	QM	0.02898	149.59
	QM/MM	0.00581	174.99
7T	QM	0.02886	149.70
	QM/MM	0.00629	173.62
8T	QM	0.02687	149.82
	QM/MM	0.00008	175.74
3T-Met	QM	0.07136	121.25
	QM/MM	0.02417	173.65
4T-Met	QM	0.07259	122.07
	QM/MM	0.00758	174.32
5T-Met	QM	0.04749	121.89
	QM/MM	0.01285	173.24
6T-Met	QM	0.07427	122.25
	QM/MM	0.00400	172.40
7T-Met	QM	0.03871	122.52
	QM/MM	0.00010	174.62
8T-Met	QM	0.07546	122.32
	QM/MM	0.00854	176.07
3T-Eth	QM	0.12960	115.31
	QM/MM	0.01145	176.30
4T-Eth	QM	0.14049	118.29
	QM/MM	0.02314	169.33
5T-Eth	QM	0.15070	123.05
	QM/MM	0.05283	174.91
6T-Eth	QM	0.09435	111.82
	QM/MM	0.00714	154.58
7T-Eth	QM	0.09893	111.98
	QM/MM	0.01247	173.33
8T-Eth	QM	0.10528	114.23
	QM/MM	0.01983	174.78

**Table 2:** Reorganization energy for non-, methyl-, and ethyl-substituted 3T–8T P3AT from both QM and QM/MM calculations.

System	Non-		Methyl- λ_QM/MM_ (kcal/mol)	Ethyl- λ_QM/MM_ (kcal/mol)
	λ_QM_ (kcal/mol)	λ_QM/MM_ (kcal/mol)		
3T	10.148	9.849	12.144	13.444
4T	9.479	7.680	12.143	13.015
5T	9.067	7.455	10.862	11.074
6T	8.539	6.867	10.097	10.247
7T	8.029	6.945	9.734	8.593
8T	7.558	6.086	7.084	7.474
